# The pathogenic role of connective tissue growth factor in osteoarthritis

**DOI:** 10.1042/BSR20191374

**Published:** 2019-07-19

**Authors:** Min Tu, Yao Yao, Feng Hua Qiao, Li Wang

**Affiliations:** 1 Department of Orthopedics, Second People’s Hospital of Jingmen, Jingmen 448000, China; 2 Department of Gastrointestinal Surgery, Second People’s Hospital of Jingmen, Jingmen 448000, China; 3 Department of Gynecology, Second People’s Hospital of Jingmen, Jingmen 448000, China; 4 Department of Gynecology, First People’s Hospital of Jingmen, Jingmen, HuBei 448000, China

**Keywords:** cartilage homeostasis, connective tissue growth factor, inflammatory mediators, Osteoarthritis

## Abstract

Osteoarthritis (OA) is the most common form of arthritis, and connective tissue growth factor (CTGF) is found to be up-regulated in adjacent areas of cartilage surface damage. CTGF is present in osteophytes of late stage OA. In the present study, we have reviewed association of CTGF in the development and progression of OA and the potential effects of CTGF as a therapeutic agent for the treatment of OA. We have reviewed the recent articles on CTGF and OA in databases like PubMed, google scholar, and SCOPUS and collected the information for the articles. CTGF is usually up-regulated in synovial fluid of OA that stimulates the production of inflammatory cytokines. CTGF also activates nuclear factor-κB, increases the production of chemokines and cytokines, and up-regulates matrix metalloproteinases-3 (MMP-3) that in turn leads to the reduction in proteoglycan contents in joint cartilage. Consequently, cartilage homeostasis is imbalanced that might contribute to the pathogenesis of OA by developing synovial inflammation and cartilage degradation. CTGF might serve as a useful biomarker for the prognosis and treatment of OA, and recent studies have taken attempt to use CTGF as therapeutic target of OA. However, more investigations with clinical trials are necessary to validate the possibility of use of CTGF as a biomarker in OA diagnosis and therapeutic target for OA treatment.

## Introduction

**Figure 1:**
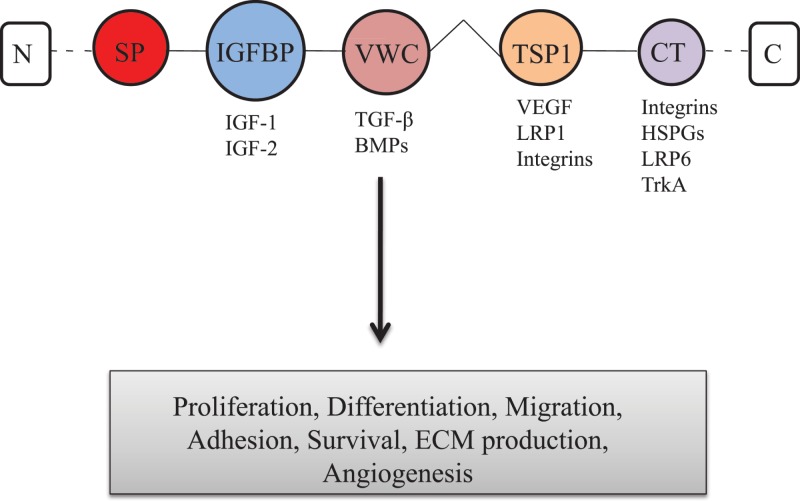
CTGF protein structure CTGF have five domains, including secretory SP, IGFBP, VWC, TSP1, and C-terminal cystine-knot (CT) modules. Some of the known binding partners of each module are also listed: integrins; IGFs; bone morphogenic proteins; transforming growth factor-β (TGF-β); LRP1; VEGF; HSPGs; TrkA [7–9,13].

Connective tissue growth factor (CTGF; also known as CCN2) is a member of the CCN (CYR61-CTGF-NOV) family, which is a group of secreted multifunctional proteins [1–3]. CTGF is known to be up-regulated in pathological conditions in regions of severe injury including fibrotic disorders, various cancers, and arthritis. CTGF has also been shown to be up-regulated adjacent to areas of cartilage surface damage, and present and in osteophytes of late stage osteoarthritis (OA) [4–6]. CTGF was first discovered by Bradham in 1991. It is a 38 kD cysteine-rich (CR) protein made up of five domains, including secretory signal peptide (SP), insulin-like growth factor binding protein (IGFBP), von Willebrand factor type C repeat (VWC), thrombospondin type 1 repeat (TSP1), and C-terminal cystine-knot (CT) modules. The N-terminal domain of CTGF interacts with aggrecan and mediates myofibroblast differentiation and collagen synthesis. IGFBP binds IGFs with high affinity and exerts biological effects by modulating IGF behavior. VWC domains, also referred to as chordin-like CR repeats, are an extremely common motif found in extracellular matrix (ECM) proteins. TSP domain binds a wide range of extracellular targets and important signaling molecules including collagen V, fibronectin, TGF-β, and heparin. The CT domain interacts with members of the TGF-β superfamily and mediates fibroblast proliferation [7–9]. Figure 1 shows CTGF protein structure. This matricellular protein is required for the development of fibrotic tissues in a variety of organs. It is strongly expressed in growth of cartilage, especially in hypertrophic chondrocytes, and plays an essential role in endochondral ossification by promoting angiogenesis, proliferation, and differentiation of chondrocytes [10–12]. CTGF has also been played role in a diverse range of cellular functions including migration, adhesion, survival, wound healing, and synthesis of ECM proteins in various cell types. CTGF exerts its functions by binding to various cell surface receptors including integrin receptors, cell surface heparan sulfate proteoglycans (HSPGs), low density lipoprotein receptor-related protein (LRP), and the tyrosine kinase receptor (TrkA) [13]. OA is the most common form of arthritis, and a major cause of morbidity. It is a chronic joint disorder accompanied by varying degrees of functional limitation and reduced quality of life. OA is characterized by slow progressive degeneration of articular cartilage, fibrosis, subchondral bone alteration, osteophyte formation, and secondary induced synovitis [14–16]. OA becomes more frequent in both sexes amongst the older people, and is supposed to be the fourth leading cause of disability by the year 2020 [17,18]. The cause of the OA is unclear, although obesity, ageing, sex, genetic factors, biological, and biomechanical components have been associated with increased risk of OA. Numerous studies have suggested that increased production and activation of degradative enzymes, altered synthesis of cartilage matrix molecules, and growth factors are believed to play a central role in this pathological process [19,20]. The most common clinical symptoms of OA are joint pain, swelling, decreased range of motion and stiffness [21]. OA cannot be cured totally, only some management is purely supportive to control the symptoms and pain reduction. However, most of the conventional management options are not always satisfactory as these options are not readily available, expensive and have large risk of side effects [22,23]. To overcome these problems, development of new and potential alternative therapeutics is urgently needed. It is also important to know the factors that are involved in the pathophysiology of OA to identify the more effective targets for OA therapy [24–26].

In this review, we have focussed on the association of CTGF in the development and progression of OA and the potential effects of CTGF as a therapeutic agent for the treatment of OA.

## Abnormal CTGF expression in disease progression

CTGF plays an important role in many physiological and pathological activities including inflammation, wound-healing, fibrosis, angiogenesis, and carcinogenesis [11,27]. CTGF has been implicated in all fibrotic conditions and detected in joint capsule tissue or contracted joints of the synovium in hemophilia patients [1]. Some evidences identified that increased expression of CTGF could also be involved in the onset of rheumatoid arthritis by enhancing the activity of osteoclasts [9]. CTGF is normally expressed in developing tissues; however, it is reported to be the most abundantly expressed growth factor in chondrocytes of human patients with severe OA. CTGF mRNA has been found to be up-regulated adjacent to areas of cartilage surface damage and present in chondro-osteophytes [6]. CTGF expression is modulated by several factors, including transforming growth factor β (TGFβ), dexamethasone, macrophage colony stimulating factor, VEGF, prostaglandin E2 (PGE2), and retinoic acidin various cell types. CTGF is also up-regulated in response to mechanical stimuli [5,28]. In an animal model, CTGF overexpression in synovial lining of mouse knee joints induces fibrosis and cartilage damage. The cartilage damage could be either a direct effect of CTGF overexpression or a result of factors excreted by the CTGF-induced fibrotic synovial tissue [29].

## Role of CTGF in OA pathogenesis

**Figure 2:**
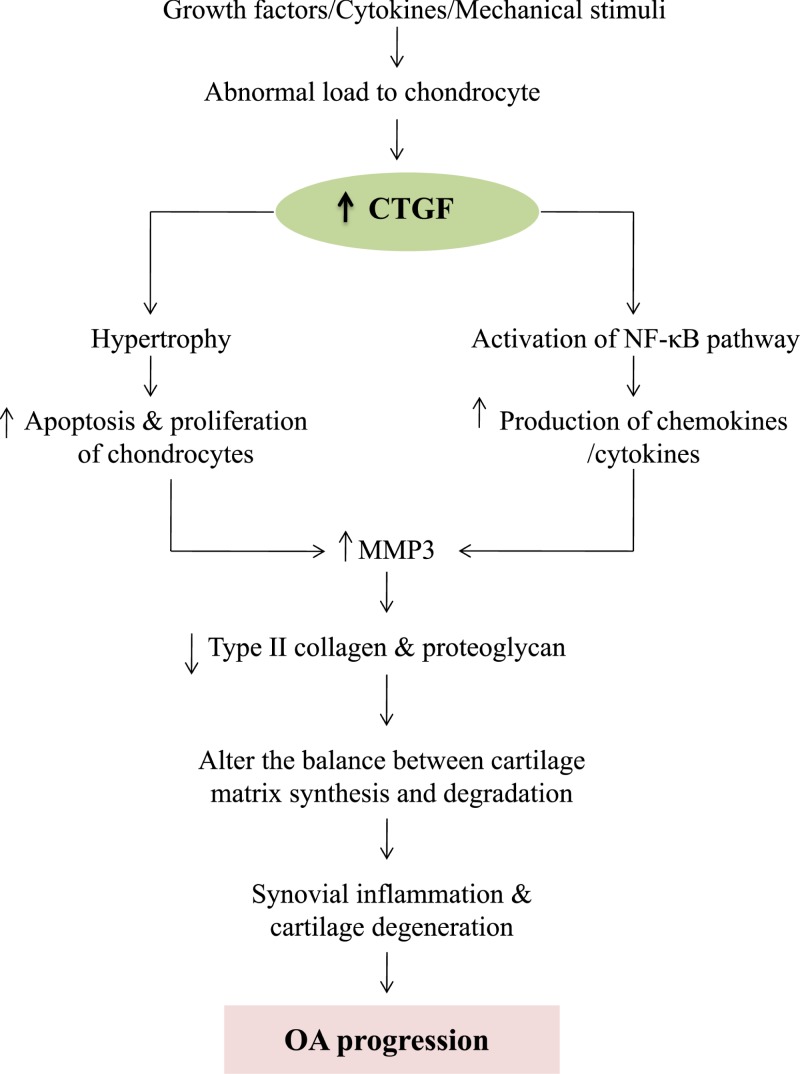
The role of CTGF in OA pathogenesis The expression of CTGF is up-regulated in synovial fluid of OA and up-regulation of CTGF stimulates the production of inflammatory cytokines [32,35,36]. CTGF also activates NF-κB pathway, increases the production of chemokines and cytokines, and up-regulates MMP-3 which in turn leads to the reduction in the content of proteoglycans in joint cartilage. As a consequence, cartilage homeostasis is imbalanced that might contribute to the pathogenesis of OA by developing synovial inflammation and cartilage degradation [29,33].

**Figure 3:**
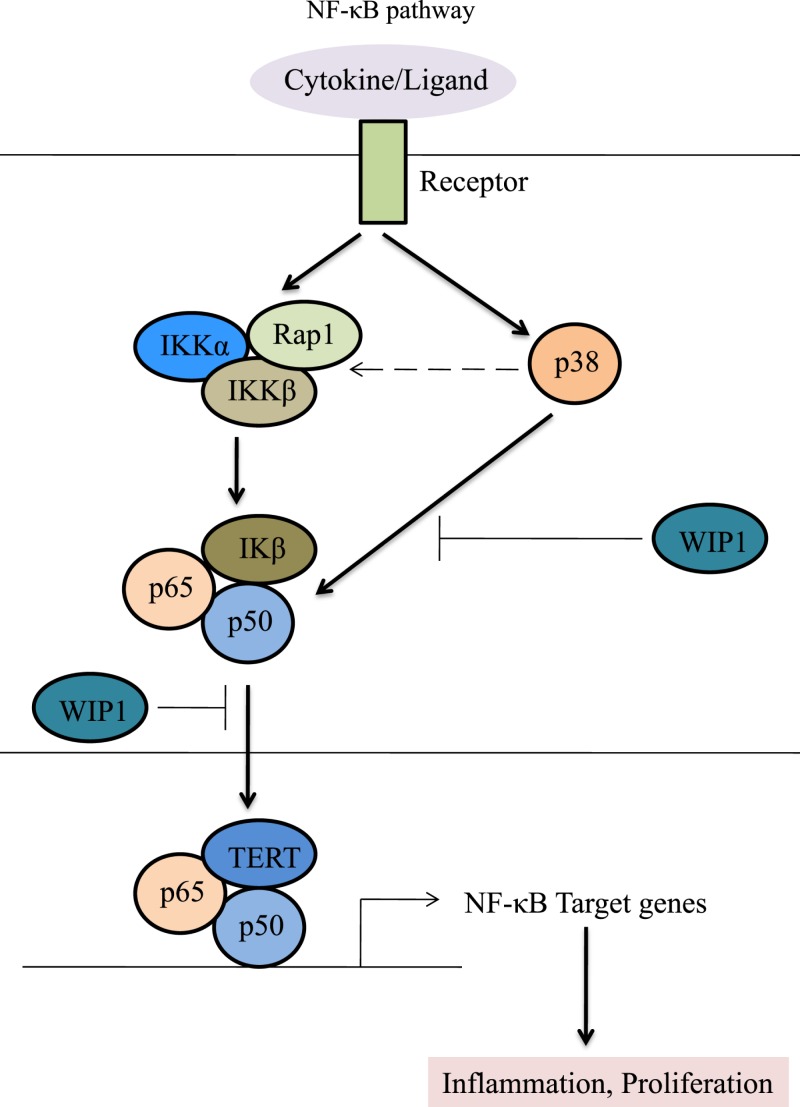
NF-κB signaling and its target genes NF-κB signaling pathway constitutes a family of transcription factors that are stimulated by pro-inflammatory cytokines or ligands. The activated NF-κB molecules trigger the expression of target genes which induce destruction of the articular joint, leading to OA progression [37–39].

**Figure 4:**
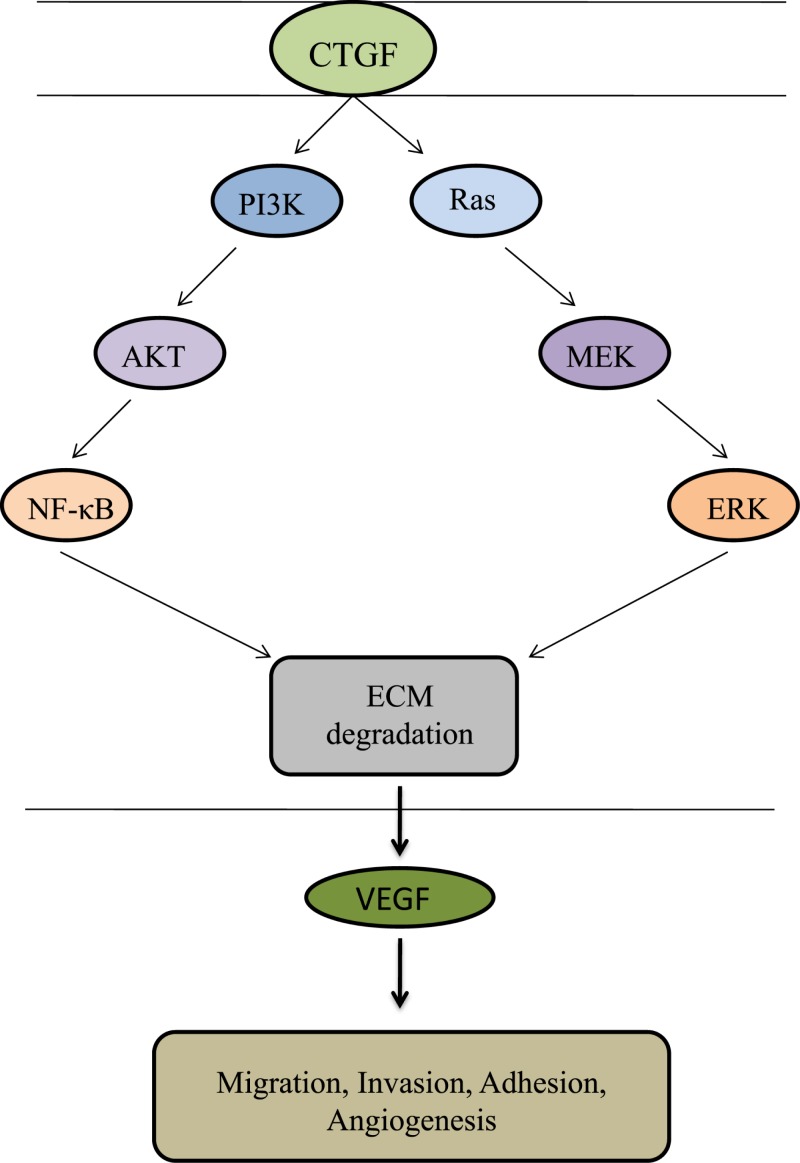
Role of CTGF in VEGF production in OA synovial fibroblasts CTGF activates PI3K, AKT, ERK, and NF-κB pathway, inducing VEGF expression and promoting angiogenesis, proliferation and chondrocyte differentiation in human synovial fibroblasts [36].

CTGF is currently an attracting attention as it has several potential functions in chondrocytes. During chondrogenesis, CTGF acts as a signal conductor in matrix remodeling and endochondral ossification by promoting angiogenesis, proliferation and chondrocyte differentiation, the expression of type II collagen, and aggrecan amongst other factors, and the activation of integrin signaling [30,31]. CTGF plays a critical role in synovial fibrosis and osteophyte formation, and has been reported to contribute to the pathogenesis of OA. Figure 2 represents the pathogenic role of CTGF in OA progression. CTGF has been found to be the most abundantly expressed growth factor in chondrocytes of human patients with severe OA [32]. CTGF can modulate the balance between synthesis and degradation of the matrix in the cartilage in inflammatory arthritis [33]. CTGF overexpression of syovium in OA patients caused by up-regulation of matrix metalloproteinase 3 (MMP-3) would reduce the content of proteoglycans in joint cartilage and result in transient fibrosis [29]. In OA knees, TGF-β stimulates CTGF expression, and is involved in joint swelling by inducing synovial cells to synthesize endogenous hyaluronan [34]. It has been demonstrated that many OA patients show a changed morphology and inflammatory phenotype in the synovial tissue characterized by hypertrophy and the infiltration of leucocytes and monocytes/macrophages. Monocyte chemoattractant protein-1 (MCP-1/CTGF) is the key chemokine that regulates migration and infiltration of monocytes, and are critical mediators of the disturbed metabolism and enhanced catabolism of joint tissue involved in OA. CTGF enhances the monocyte migration in OASFs by increasing MCP-1 expression through the αvβ5 integrin, FAK, MEK, ERK, and nuclear factor-κB (NF-κB)/AP-1 signal transduction pathway. Migration and infiltration of mononuclear cells to inflammatory sites has been reported to enhance the synovial inflammation and secreted inflammatory factors (IL-1, IL-6, IL-8, TNF-α, and PGE2) and a variety of MMP, and is thought to play a central role during OA pathogenesis [35,36]. CTGF participates in the inflammatory process in OA through NF-κB-dependent pathway. NF-κB signaling pathway constitutes a family of transcription factors that are stimulated by pro-inflammatory cytokines or ligands. NF-κB is induced by ectopic expression of Rap1 (Trf2IP, an essential modulator of NF-κB-mediated pathways) that forms a complex with IKKs. IKKs recruits to and phosphorylate the p65 subunit of NF-κB to make it transcriptionally active competent. Upon stimulation, the activated NF-κB molecules trigger the expression of target genes which induce destruction of the articular joint, leading to OA progression. WIP1 phosphatase is a negative regulator of NF-κB signaling. Overexpression of WIP1 resulted in decreased NF-κB activation, whereas WIP1 knockdown resulted in increased NF-κB function and enhanced inflammation (Figure 3) [37–39]. In addition, VEGF (a mediator of angiogenesis) acts as the potential growth factor implicated in the pathogenesis of OA. CTGF induces production of VEGF by raising miR-210 expression via PI3K, AKT, ERK, and NF-κB/ELK1 pathways, contributing to inhibit glycerol-3-phosphate dehydrogenase 1-like expression and prolyl hydroxylases-2 activity, promoting hypoxia-inducible factor-1α-dependent VEGF expression and angiogenesis in OASFs (Figure 4) [36]. Zhao et al. suggested that epigenetic changes in the methylation status of CTGF contribute to the pathology of OA. They reported that CTGF gene is hypomethylated in OA chondrocytes, and has a consistent correlation with mRNA expression [40]. CTGF promotes interleukin (IL)-1β-mediated synovial inflammation in knee OA [41]. In contrast with these studies, other experiments indicate a protective or anabolic role of CTGF in OA. CTGF is suspected to play a critical role in cartilage repair. In the MIA-induced OA model, Nishida et al. showed that the local administration of recombinant CTGF (rCTGF) into defective articular cartilage could regenerate the cartilage. They administered a single injection of rCTGF incorporated in gelatin hydrogel into the joint cavity of rats and demonstrated repair of damaged cartilage to the extent that it became histologically similar to normal articular cartilage. Therefore, CTGF expression and regulation are of particular interest [42].

## CTGF -targetted treatment approach in OA

**Figure 5:**
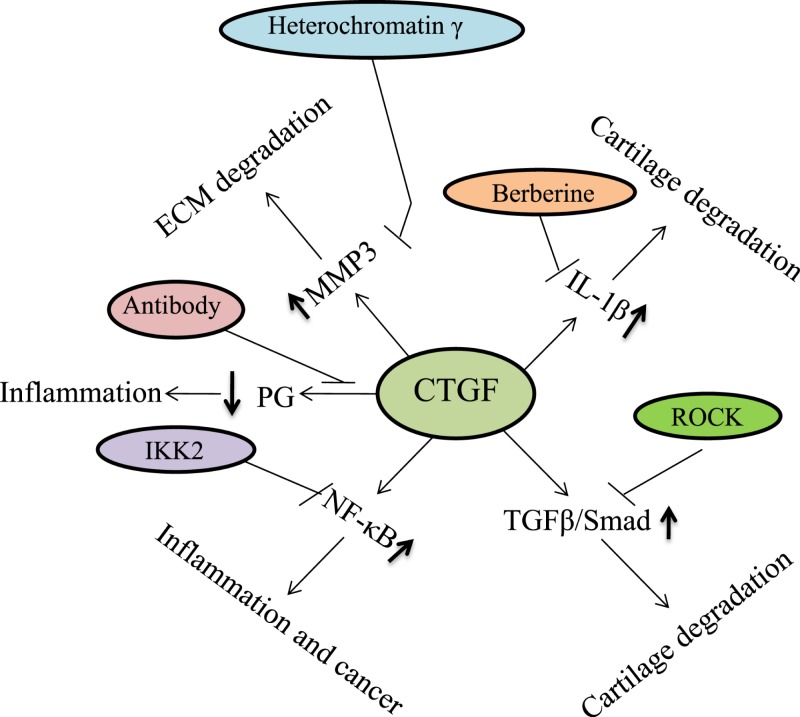
Therapeutic roles of CTGF in the treatment of OA CTGF can be a good target for the drug development to treat OA [14,43].

OA is the leading cause of morbidity and the most common form of arthritis characterized by progressive degeneration of articular cartilage. The conventional therapy of OA is still unsatisfactory. The use of biochemical markers may diagnose the disease at an earlier stage, and may develop safe and effective disease modifying treatments for patients with OA. CTGF might serve as a useful biomarker for the prognosis and treatment of OA [14,43]. Table 1 and Figure 5 summarize the therapeutic use of CTGF in the treatment of OA.

**Table 1:** Potential therapeutic roles of CTGF in the treatment of osteoarthritis

Therapeutic agents	Experimental cells/model	Effects	Ref
Berberine	Collagenase-induced rat model	Attenuated CTGF-induced IL-1β expression and exhibited an anti-inflammatory effect	[44]
αvβ5 integrin neutralized antibody and ASK1 shRNA	Human synovial fibroblasts	Attenuated CTGF mediated IL-6 production	[2]
ROCK inhibitor	Osteoarthritic articular chondrocytes	Inhibited the cartilage degradation via TGFβ /Smad signaling	[28]
Antibodies against CTGF	Chondrocyte cell lines	Stimulated PG synthesis	[45]
Heterochromatin protein gamma	Osteoarthritic articular chondrocytes	Suppressed the expression of MMP3	[46]
Harmine	Human chondrocytic HCS-2/8 cells and osteoarthritic articular chondrocytes	Showed chondroprotective effect	[48]
Thrombospondin 1 type 1 repeat module (TSP1)	Osteoarthritic articular chondrocytes and rat model	Induces cartilage regeneration	[49]

A significant number of natural compounds that induce CTGF in chondrocytes could be novel candidates to correct cartilage degenerative changes in OA. Berberine has been suggested as a novel therapeutic strategy for managing OA. CTGF-induced IL-1β expression in OASFs is attenuated by the treatment with berberine. Berberine exhibits an anti-inflammatory effect which reverses cartilage damage in an experimental model of collagenase-induced OA. Berberine was found to inhibit the signaling components in OASFs *in vitro* and prevent cartilage degradation *in vivo* [44]. OASFs stimulation with CTGF induced concentration-dependent increases in IL-6 expression. CTGF mediated IL-6 production was attenuated by αvβ5 integrin neutralized antibody and apoptosis signal-regulating kinase 1 (ASK1) shRNA. The potentiating action of CTGF can also be inhibited by treating with p38 inhibitor (SB203580), JNK inhibitor (SP600125), AP-1 inhibitors (Curcumin and Tanshinone IIA), and NF-κB inhibitors (PDTC and TPCK) [2]. Woods et al. showed that CTGF is responsive to both Rac1 and actin pathways in chondrocytes which might be relevant to OA. They suggested that CTGF expression might be controlled by either inhibition of actin polymerization (cytochalasin D treatment), promotion of actin polymerization (jasplakinolide treatment), inhibition of RhoA/rho kinase (ROCK) signaling (Y27632 treatment), and Rac1 signaling [28]. CTGF is involved in inhibition of PG synthesis. Minato et al. established three different antibodies against CTGF that stimulated PG synthesis in chondrocyte cell lines [45]. Masuko et al. obtained articular cartilage samples from OA patients and chondrocytes were isolated and cultured *in vitro*. They stimulated chondrocytes with PGE2, PGE receptor (EP)-specific agonists/IL-1. They showed that stimulation of chondrocytes with PGE2 or IL-1 significantly suppressed CTGF expression [5].

Matrix metalloproteinase 3 (MMP3) is well known as a secretory endopeptidase that also acts as a trans regulator of CTGF. CTGF is activated by overexpressed MMP3 and knocking down of MMP3 suppressed CTGF expression [46]. Eguchi et al. suggested that the role of MMP3 in the development, tissue remodeling, and pathology of arthritic diseases can be regulated through CTGF. They determined that heterochromatin protein γ coordinately regulates CTGF by interacting with MMP3. The potentially therapeutic effect of intact CTGF on cartilage regeneration has been indicated by a number of studies [46]. In a study, Tang et al. determined the function of CTGF in OA development. They generated a postnatal, conditional CtgfcKO mouse for cartilage injury experiments and to explore the course of OA. They reported that deletion of CTGF *in vivo* increased the thickness of the articular cartilage and protected mice from OA through TGF-β-dependent SMAD2 phosphorylation [47].

In contrary, there are evidences that CTGF inducer may also have beneficial effects on OA treatment. Hara et al. suggested that a significant number of natural compounds that induce CTGF in chondrocytes could be novel candidates to correct cartilage degenerative changes incurred in OA. They identified the β-carboline alkaloid harmine as a novel inducer of CTGF in human chondrocytic HCS-2/8 cells and osteoarthritic articular chondrocytes. The chondroprotective effect of harmine was investigated by stimulation with TNFα, and it was shown to ameliorate TNFα-induced decrease in expression of CTGF and cartilage markers aggrecan, SOX-9 and COL2α1 [48]. Abd El Kader et al. suggested thrombospondin 1 type 1 repeat module (TSP1) as a promising regenerative therapeutics of OA. Their study showed that TSP1 displayed more prominent regenerative effects than intact CTGF on damaged cartilage [49]. There is a lack of evidence that may support that reducing the expression of CTGF is a successful strategy in OA treatment. More investigations are necessary to check the performance of CTGF in a specific cohort of patients with earlier stage. It is also essential to define the signaling events induced by CTGF for the inhibition of CTGF-mediated inflammatory process.

Inhibitors of NF-κB pathway signaling might be used for a variety of autoimmune and inflammatory disorders. Ikk2 (IκB kinase) is a component of NF-κB pathway that is important drug target both in inflammation and cancer. Ikk2 coordinates the cellular response to a diverse set of extracellular stimuli. In response to an external stimulus, Ikk2 induces NF-κB transcription factor, which activates the transcription of genes that regulate a variety of fundamental biological processes. Targetting this kinase by inhibitors may underscore the potential for therapeutic intervention. VEGFR inhibitors might also be used for treating inflammation and cancer by targetting anti-angiogenic mediators. γ-tocotrienol (a vitamin E derivative derived from palm oil) inhibited VEGF- induced migration, invasion, tube formation and viability of HUVECs *in vitro* as well as reduced the blood vessels formation. γ-tocotrienol was also found to inhibit angiogenesis-dependent growth of human hepatocellular carcinoma through abrogation of AKT/mTOR pathway in an orthotopic mouse model. Noncoding RNAs (ncRNA) might play an important role in the regulation of inflammatory signaling. The use of ncRNA may open new avenues for the potential therapeutic intervention in inflammatory diseases, from arthritis to cancer, which may be translated into clinical outcomes in the future [50–52].

## Conclusion

OA is becoming a big threat for normal healthy life. Early diagnosis and administration of effective treatment may be the best strategies against OA. Recent studies have taken attempt to use CTGF as a diagnostic marker for OA to evaluate its treatment effects. More investigations with clinical trials are necessary to validate the possibility of use of CTGF as a biomarker in OA diagnosis. The use of CTGF-antagonists can be a good candidate for the drug development to treat OA in near future. The better understanding of the role of CTGF in OA may provide a basis for new therapeutic approaches to treat OA.

## Abbreviations

CR: cysteine-rich
CTGF: connective tissue growth factor
ECM: extracellular matrix
HSPG: heparan sulfate proteoglycan
IGFBP: insulin-like growth factor binding protein
IGF: insulin-like growth factor
IL: interleukin
LRP1: lipoprotein receptor-related protein1
MCP-1: monocyte chemoattractant protein-1
MCSF: macrophage colony stimulating factor
MMP: matrix metalloproteinases
NF: nuclear factor
OA: osteoarthritis
PGE2: prostaglandin E2
SP: signal peptide
TSP1: thrombospondin type 1 repeat
VEGF: vascular endothelial growth factor
VWC: von Willebrand factor type C
